# Effects of cold water immersion and compression garment use after eccentric exercise on recovery

**DOI:** 10.20463/jenb.2019.0007

**Published:** 2019-03-31

**Authors:** Tatsuhiro Maruyama, Sahiro Mizuno, Kazushige Goto

**Affiliations:** 1 Graduate School of Sports and Health Science, Ritsumeikan University, Kusatsushi, Shiga Japan

**Keywords:** recovery, eccentric exercise, muscle damage

## Abstract

**[Purpose]:**

The combined effect of different types of post-exercise treatment has not been fully explored. We investigated the effect of combined cold water immersion (CWI) and compression garment (CG) use after maximal eccentric exercise on maximal muscle strength, indirect muscle damage markers in the blood, muscle thickness, and muscle soreness score 24 h after exercise.

**[Methods]:**

Ten men performed two trials (CWI + CG and CON) in random order. In the CWI + CG trial, the subjects performed 15 min of CWI (15°C), followed by wearing of a lower-body CG for 24 h after exercise. In the CON trial, there was no post-exercise treatment. The exercise consisted of 6 × 10 maximal isokinetic (60°·s^-1^) eccentric knee extensions using one lower limb. The maximal voluntary contraction (MVC) and maximal isokinetic (60°·s^-1^) strength during knee extension, as well as the indirect muscle damage markers, were evaluated before exercise and 24 h after exercise.

**[Results]:**

The maximal muscle strength decreased in both trials (*p* < 0.001), with no difference between them. The exercise-induced elevation in the myoglobin concentration tended to be lower in the CWI + CG trial than in the CON trial (*p* = 0.060). The difference in the MVC, maximal isokinetic strength, muscle thickness, and muscle soreness score between the trials was not significant.

**[Conclusion]:**

CWI followed by wearing of a CG after maximal eccentric exercise tended to attenuate the exercise-induced elevation of indirect muscle damage markers in the blood.

## INTRODUCTION

Eccentric muscle contraction during training or competition causes exercise-induced muscle damage^1^^,^^2^, which is characterized by increases in the serum creatine kinase (CK) and myoglobin (Mb) concentrations, delayed-onset muscle soreness, swelling, and decreased maximal muscle strength^3^^-^^5^. In competitive athletes under training situations, rapid recovery of exercise performance after exercise is important in improving performance during the next training session and in preventing excessive fatigue associated with overtraining.

Although several recovery strategies, including massage^6^, cryotherapy^7^, active recovery^8^, stretching^9^, and nutritional supplementation^10^, are currently advocated, the most prevalent post-exercise treatment is cold water immersion (CWI)^7^. Post-exercise CWI attenuates exercise-induced increases in swelling, inflammation, fatigue, and soreness^11^^,^^12^. CWI at 10°C for 10 min following 90 min of intermittent shuttle testing significantly attenuated the decrease in the maximal voluntary contraction (MVC) for knee flexion, elevations in the serum Mb concentration, and soreness scores within 48 h post-exercise^13^. However, there has been an increasing interest in the use of a compression garment (CG) as a novel post-exercise treatment. Wearing a whole-body CG within 24 h following resistance exercise significantly promoted the recovery of maximal strength in the upper and lower limb muscles^14^. Moreover, it significantly promoted the recovery of maximal power output for bench throw exercises, in addition to reducing the degree of muscle swelling and muscle soreness^15^. Furthermore, wearing of a lower-limb CG for 12 h following 100 plyometric drop jumps promoted the recovery of jump performance^16^.

Despite extensive evidence of the improved recovery of muscle function with the use of a CG, the combined effect of different types of post-exercise treatment has not been fully explored. This topic would be of practical interest because athletes and coaches generally apply multiple treatments to maximize recovery after exercise. Furthermore, athletes are required to train every day. We believe that recovery during the initial 24 h after exercise is the most important. Therefore, we investigated the effect of combined CWI and CG use after maximal eccentric exercise on maximal muscle strength, indirect muscle damage and inflammation markers in the blood, muscle swelling, and muscle soreness score within 24 h post-exercise in the present study. We hypothesized that CWI followed by wearing of a CG after maximal eccentric exercise would improve the recovery of the MVC and attenuate exercise-induced elevations in the CK, Mb, and high-sensitive C-reactive protein (hsCRP) concentrations.

## METHODS

### Subjects

Ten healthy men [mean ± standard error (SE): age, 22.3 ± 0.7 years; height, 173.9 ± 1.5 cm; body mass, 67.6 ± 2.1 kg; body mass index, 22.4 ± 0.5 kg·m^-2^] participated in the study. All of them were physically active and had several years of experience in performing sports (exercising approximately 3 days per week). None of them were involved in a regular training program at the start of the study. The subjects were prohibited from drinking alcoholic beverages at least 24 h prior to the measurements. After being informed on the purpose of the present study, all subjects provided written informed consent. The study was approved by the Ethics Committee of Ritsumeikan University, Japan.

### Experimental design

**Figure 1:**
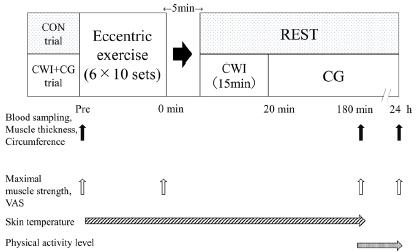
Experimental design for the main experiments

All subjects visited the laboratory five times during the experimental period. On the first visit, they were familiarized with the exercise session and measurements. On the second and third visits, the main experiment for the first trial was conducted, and on the fourth and fifth visits, the main experiment for the second trial was conducted. The main experiment consisted of two trials (CWI + CG or CON) in a randomized crossover design. In both trials, the subjects initially conducted 60 bouts of maximal eccentric contraction of the quadriceps femoris muscle. In the CWI + CG trial, the subjects immersed their lower-body muscles in cold water (15°C) for 15 min and then wore a CG during the remainder of the 24 h post-exercise. In the CON trial, the subjects did not conduct CWI nor wear a CG post-exercise. The two trials were separated by more than a week. Changes in the muscle strength and the concentrations of the indirect muscle damage markers within 24 h post-exercise were compared between the two trials (Figure 1).

### Maximal eccentric exercise

Exercise-induced muscle damage was induced with 60 bouts of maximal eccentric contraction of the quadriceps femoris muscle. The exercise consisted of 60 (6 × 10 sets) maximal isokinetic (60°·s^-1^) eccentric contractions via unilateral knee extension. A 60-s rest period was allowed between sets. During eccentric contraction, the subjects were verbally encouraged to resist the motion of the lever arm maximally throughout the prescribed range of motion [from 180° (fully extended position) to 90° (flexed position)] with a constant velocity of 60°·s^-1^, using an isokinetic dynamometer (Biodex system 4; SAKAI Medical Co., Tokyo, Japan). The peak torque during each contraction was measured to evaluate the work volume during the 60 bouts of eccentric contraction.

### CWI

In the CWI + CG trial, the lower-limb muscles were immersed in a water pool (INTEX; Onda Co., Tokyo, Japan; 122 × 122 × 30 cm) for 15 min. The water temperature was maintained at 15°C. During CWI, the subjects rested in an extended sitting position, with both lower limbs entirely immersed in the water. CWI was started 5 min after completion of the eccentric exercise. In the CON trial, the subjects rested by sitting on a chair for 15 min.

### CG

In the CWI + CG trial, the subjects started wearing a lower-body CG (UA Recharge Energy Leggings; Under Armour, Baltimore, MD, USA) after completing 15 min of CWI. The appropriate size of the CG was selected on the basis of the subjects’ body height and waist circumference. The subjects wore the CG until 24 h after the completion of the exercise, including during their sleep, except when they showered.

### Measurements

#### Maximal muscle strength

An isokinetic dynamometer (Biodex system 4; SAKAI Medical Co.) was used to evaluate the MVC and maximal isokinetic strength during knee extension before exercise, immediately after exercise, and 3 h and 24 h after exercise. The MVC was measured at a knee angle of 105° (full lower limb extension, defined as 180°), with all subjects requested to exert maximal strength for 3 s. The MVC measurements were repeated twice, and the highest value was recorded. The maximal isokinetic strength for knee extension was measured at an angular velocity of 60°·s^-1^. The subjects performed three bouts of isokinetic knee extension, and the highest value among the three repetitions was recorded.

#### Muscle thickness and thigh circumference

The thickness of the vastus lateralis muscle was measured at the midpoint (50% of the distance between the greater trochanter and patellar tendon) of the thigh using an ultrasound system (IPC-1531; Aloka, Tokyo, Japan), as the exercise protocol mainly recruited the quadriceps muscle. The thigh circumference was measured at the same point using a tape measure. The point was marked to ensure measurements at the same place. The measurements were repeated before exercise and 3 h and 24 h after exercise.

#### Skin temperature, heart rate (HR), and physical activity

Skin temperature was assessed at the midpoint of the thigh (50% of the distance between the greater trochanter and patellar tendon) using an NT logger (NIKKISO-THERM, Tokyo, Japan) and HR using a wireless HR monitor (Acculex Plus; Polar Electro Oy, Kempele, Finland). Skin temperature and HR were measured continuously for 3 h after exercise, with data collected every 1 min. Post-exercise HRs reflect sympathetic and parasympathetic nerve activities at rest^17^. Physical activity was evaluated between 3 h and 24 h after exercise using an acceleration sensor (Actimaker; Panasonic Electric Works Co., Osaka, Japan).

#### Muscle soreness score

Subjective muscle soreness was scored using a 100-mm visual analog scale before exercise, immediately after exercise, and 3 h and 24 h after exercise; 0 mm indicates no pain and 100 mm the worst pain imaginable^18^.

### Blood sampling and analysis

Blood samples from the antecubital vein were collected before exercise and 3 h and 24 h after exercise to measure the blood lactate, glucose, serum CK and Mb, and hsCRP concentrations.

Serum and plasma were obtained via centrifugation of the blood samples for 10 min at 4°C (3,000 revolutions per min) and then stored at −60°C until the analysis. The blood glucose and lactate concentrations were measured using a glucose analyzer (Flee style; Nipro Co., Osaka, Japan) and a lactate analyzer (Lactate Pro; Arkray Co., Kyoto, Japan), respectively, immediately after blood collection. The serum CK, Mb, and hsCRP concentrations were measured at the SRL Clinical Laboratory (Tokyo, Japan). The intra-assay coefficient of variation for each measurement was 4.0% for CK, 2.0 % for Mb, and 2.7% for hsCRP.

### Statistical analysis

Data were expressed as means ± SEs. Changes in the muscle strength, thigh circumference, muscle thickness, skin temperature, HR, muscle soreness score, and blood variables over time were compared using two-way analysis of variance (ANOVA) with repeated measures. When the ANOVA revealed a significant interaction (trial × time) or main effects (trial and time), the Tukey-Kramer post-hoc test was performed to identify significant differences. The area under the curve for the CK and Mb concentrations was compared using the paired t-test. For all tests, a *p*-value of < 0.05 was considered to indicate statistical significance.

## RESULTS

The total work volume during 60 bouts of maximal eccentric contraction was not significantly different between the CWI + CG (1588 ± 30 J) and CON trials (1716 ± 38 J, *p* = 0.092, *F* = 3.555). There were also no significant differences in the average torque (290 ± 3 Nm and 297 ± 4 Nm, respectively; *p* = 0.617, *F* = 0.268) or energy expenditure after exercise [from 3 h to 24 h after exercise completion: 1850 ± 61 kcal and 1847 ± 61 kcal; p = 0.927, t(9) = 0.094].

### Skin temperature and HR

**Figure 2:**
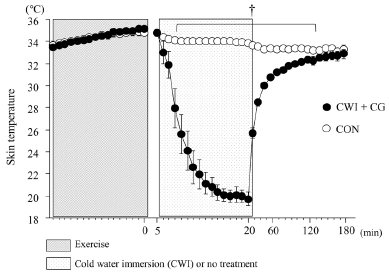
Skin temperature during exercise and 3 h after exercise. Values are presented as means ± standard errors. ^†^*p* < 0.05 between the two trials

Before the start of exercise, the skin temperature did not differ significantly between the two trials. In the CWI + CG trial, a rapid reduction in the skin temperature (from 35.2 ± 0.4°C to 19.7 ± 1.8°C) was observed following 15 min of CWI. Consequently, the differences between the two trials were significant from 20 min to 150 min after exercise (*p* < 0.001, *F* = 123.946, Figure 2).

**Figure 3:**
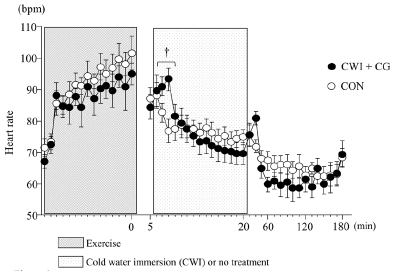
Heart rate during exercise and 3 h after exercise. Values are presented as means ± standard errors. ^†^*p* < 0.05 between the two trials

The HR increased significantly during exercise (main effect for time, p < 0.001); however, the difference between the two trials was not significant. Moreover, the HR in the CWI + CG trial was significantly higher than that in the CON trial 2 and 3 min after water immersion (interaction, *p* < 0.001, *F* = 2.458, Figure 3).

### Maximal muscle strength

**Figure 4:**
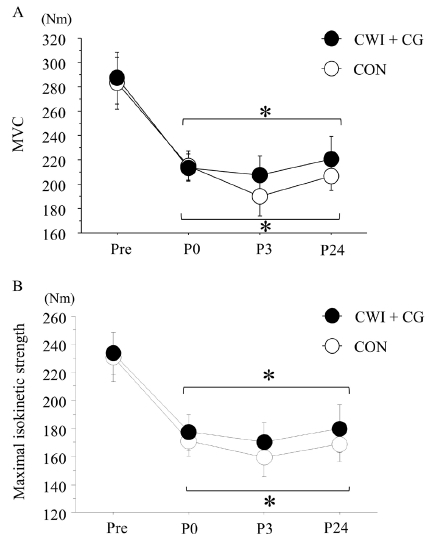
Maximal voluntary contraction (MVC) (A) and maximal isokinetic (60°·s^-1^) strength during knee extension (B) before exercise and after exercise. Values are presented as means ± standard errors. *p < 0.05 vs. pre-exercise value

Figure 4 shows the changes in the MVC and maximal isokinetic strength during knee extension. The exercise significantly decreased both the MVC and maximal isokinetic strength (MVC, *p* < 0.001, *F* = 22.002; maximal isokinetic strength, *p* < 0.001, *F* = 20.801). However, the changes in either parameter did not differ significantly between the two trials.

### Blood variables

Table 1 presents the changes in the blood variables. The blood glucose and lactate concentrations did not show a significant interaction or main effect for trial and time (blood glucose concentration: interaction, *p* = 0.490, *F* = 0.742; main effect for trial, *p* = 0.205, *F* = 1.868; main effect for time, *p* = 0.023, *F* = 4.653; blood lactate concentration: interaction, *p* = 0.306, *F* = 1.198; main effect for trial, *p* = 0.911, *F* = 0.013; main effect for time, *p* = 0.081, *F* = 2.907). The serum CK concentration significantly increased 3 h and 24 h after exercise (main effect for time, *p* = 0.045, *F* =5.387), but without a significant difference between the trials (*p* = 0.493, *F* = 0.510). The serum Mb concentration significantly increased 3 h after exercise in both trials (*p* < 0.001, *F* = 49.104); however, the exercise-induced Mb concentration elevation tended to be lower in the CWI + CG trial (81.6 ± 6.1 ng/mL) than in the CON trial (114.5 ± 15.4 ng/mL; interaction, *p* = 0.069, *F* = 4.186; main effect for trial, *p* = 0.060, *F* = 4.60). The serum hsCRP concentration was not significantly different between the two trials at any time point (main effect for time, *p* = 0.098, *F* = 3.255; main effect for trial, *p* = 0.124, *F* = 2.875).

**Table 1:** Changes in blood variables before exercise and during post-exercise (n = 10)

		Pre	3 h	24 h	Interaction (Trial×Time)	Main effect	
						Trial	Time
Glucose (mg/dL)	CWI +CG	88±2	86±2	91±2.4	0.49	0.205	0.023
	CON	91±2	87±2	91±1.6			
Lactate (mmol/L)	CWI +CG	1.4±0.2	1.5±0.1	1.4±0.1	0.306	0.911	0.081
	CON	1.2±0.1	1.4±0.1	1.6±0.2			
Mb (ng/mL)	CWI +CG	32.8±1.5	81.6±6.1^*^	35.1±1.3	0.06	0.069	< 0.001
	CON	31.5±1.8	114.5±15.4^*^	40.3±3.4			
CK (U/L)	CWI +CG	199±39	224±35	247±30	0.458	0.493	0.045
	CON	209±55	245±51	315±48			
hsCRP (ng/mL)	CWI +CG	283±126	267±122	227±77	0.279	0.943	0.69
	CON	264±81	257±75	281±62			

Values are mean ± SE, *; *P* < 0.05 vs. Pre. Mb: myoglobin, CK: creatine kinase, hsCRP: high-sensitive C reactive protein

### Muscle thickness and thigh circumference

The muscle thickness tended to increase 24 h after exercise in the CON trial [from 5.43 cm to 5.69 cm (*p* = 0.054) vs. the pre-exercise value], but not in the CWI + CG trial [from 5.58 cm to 5.61 cm (*p* = 0.632) vs. the pre-exercise value]. However, the difference between the two trials at any time point was not significant (main effect for trial, *p* = 0.966, *F* = 0.002, Table 2). The thigh circumference also did not significantly differ between the two trials at any time point (main effect for trial, *p* = 0.742, *F* = 0.116, Table 2 ).

**Table 2:** Changes in muscle thickness, thigh circumference, and muscle soreness before exercise and during post-exercise (n = 10)

		Pre	0 h	3 h	24 h	Interaction (Trial×Time)	Main effect	
							Trial	Time
Muscle thickness (cm)	CWI+CG	5.6±0.2		5.5±0.2	5.6±0.1	0.100	0.966	0.023
	CON	5.4±0.2		5.5±0.2	5.7±0.2^*^			
Thigh circumference (cm)	CWI+CG	52.8±1.3		52.4±1.3^*^	52.6±1.4	0.032	0.742	0.004
	CON	52.6±1.4		52.5±1.4	53±1.4^**^			
Muscle soreness score (mm)	CWI+CG	2.6±1.8	11.2±3	16.7±5.3	25.3±8^**^	0.352	0.018	0.712
	CON	0.9±0.2	11.9±5.3	21.2±6.7^*^	23±7.3			

Values are mean ± SE, *; *P* < 0.05 vs. Pre. **; *P* < 0.05 vs. 3h.

### Muscle soreness score

The subjective muscle soreness score was significantly elevated 24 h after exercise in both trials (*p* = 0.018, *F* = 6.807); however, the difference between the trials was not significant (*p* = 0.712, *F* = 0.145, Table 2 ).

## DISCUSSION

The present study investigated the effect of combined CWI and CG use after maximal eccentric exercise on maximal muscle strength, indirect muscle damage and inflammation markers in the blood, muscle swelling, and muscle soreness score. The results showed that CWI followed by wearing of a lower-limb CG after exercise did not promote the recovery of muscle strength. Furthermore, the changes in the muscle thickness and the CK concentration were not different between the trials, whereas the exercise-induced increase in the Mb concentration tended to be attenuated by the combined treatments after exercise.

The total work volume during 60 bouts of maximal eccentric contraction (knee extension exercise) was not significantly different between the CWI + CG and CON trials. Moreover, the magnitude of the reduction in the maximal muscle strength immediately after exercise did not differ significantly between them. Therefore, the maximal eccentric exercise caused similar levels of muscle fatigue for the quadriceps femoris muscle between the two trials. The repetition of maximal eccentric exercises by the same muscles attenuates exercise-induced muscle damage during a second bout of the same exercise (repeated bout effect)^19^. Therefore, by conducting unilateral maximal eccentric exercises using the opposite lower limb in each trial, the influence of the repeated bout effect would be successfully minimized.

As expected, the serum Mb concentration (an indirect muscle damage marker) was significantly elevated after maximal eccentric exercise, consistent with previous reports^4^^,^^20^^,^^21^. However, the exercise-induced elevation of the serum Mb concentration tended to be lower in the CWI + CG trial than in the CON trials. CWI (10°C for 10 min) decreased the post-exercise elevation of the serum Mb concentration following 90 min of intermittent shuttle testing^14^, as well as the significant Mb concentration elevation following resistance exercise^15^. A key stimulus of the decrease in muscle damage (attenuated Mb concentration elevation) by post-exercise CWI is a reduction in the skin or muscle temperature^18^. According to Roberts et al., CWI at 10°C for 10 min after resistance exercise markedly lowered the muscle temperature (by ~10°C) and skin temperature (by ~15°C)^18^^,^^22^. The similar reduction in the skin temperature in the present study suggests a similar reduction in the muscle temperature; however, the latter was not determined herein. A lower muscle temperature following CWI decreases the blood flow in the muscles, mediated by vasoconstriction^23^^-^^25^. As intramuscular metabolic activation is closely associated with blood flow in the muscles^26^^,^^27^, CWI may attenuate the initiation of processes leading to secondary muscle damage after exercise^15^^,^^28^.

The potential efficacy of a CG during/after exercise has been gaining interest. Kraemer et al. reported that wearing a whole-body CG during the first 24 h after resistance exercise attenuated elevations in the CK concentration^17^. In addition, elevated CK concentrations after arm curl exercise were significantly attenuated by wearing compressive arm sleeves^29^. The sustained pressure on the damaged muscles improves the removal of metabolites and inflammatory substances^29^ and is thought to be the mechanism underlying the attenuation of muscle damage by a CG. Therefore, this mechanism differs from that of CWI; this suggests synergistic or additive effects of CWI and CG use in attenuating post-exercise elevations in the serum Mb concentration. However, in the present study, the combination of CWI and CG use after exercise did not affect the serum CK and hsCRP concentrations. Both Mb and CK are widely used as indirect markers of muscle damage; however, the time course in their concentrations is different, with CK responding more slowly owing to its larger molecular weight^11^^,^^14^^,^^28^. Therefore, changes in the CK concentration until 72 h must be evaluated in the future. Moreover, the hsCRP concentration (an inflammatory marker in the blood) did not differ significantly between the two trials. The lack of differences in the CK and hsCRP concentrations may be attributed to the relatively small muscle volume recruited for the present exercise (only unilateral quadriceps femoris muscle).

The changes in the MVC and maximal isokinetic strength did not significantly differ between the CWI + CG and CON trials. Although two previous studies^14^^,^^16^ have demonstrated that both CWI and CG use after exercise promoted the recovery of the MVC in the lower-limb muscles, this result was not confirmed in other studies^30^^,^^31^. MVC recovery is related to a reduction in muscle swelling^32^. In the present study, because the post-exercise muscle thickness did not differ significantly between the two trials, a similar recovery of the MVC might be reasonable.

The present study had several limitations. First, we were unable to measure the concentrations of lactate or indirect muscle damage markers in the blood immediately after exercise because all subjects were required to start the post-exercise treatment from 5 min after completing the exercise. However, as the maximal muscle strength decreased immediately after exercise (~20% of reduction), the eccentric exercise apparently caused muscle fatigue. Second, owing to the experimental design employed to focus on the effect of a combination of two different treatments, we were unable to distinguish the impact of CWI from that of CG use on physiological variables; thus, the effect of either CWI alone or CG use alone has not been clarified. However, in sports, athletes typically undergo several types of post-exercise treatment (e.g., cryotherapy, wearing of a CG, massage, active rest, and nutritional treatment) to maximize recovery. Therefore, we focused on the combined treatment effects rather than single-treatment effects after exercise. Finally, we collected all data from healthy adults, not from athletes. Thus, further studies are required to confirm that the present findings are also applicable among athletes.

In conclusion, CWI followed by wearing of a lower-limb CG after exercise did not promote a higher recovery of muscle strength or affect muscle thickness (as an indication of swelling). However, the exercise-induced increases in the serum Mb concentration (as an indirect muscle damage marker) tended to be attenuated by the combined use of CWI and a CG. Our findings demonstrate the potential efficacy of these two treatments in attenuating muscle damage.
